# An exploration of the relationship between active learning and student motivation in STEM: a mixed methods study

**DOI:** 10.1152/advan.00247.2022

**Published:** 2024-06-27

**Authors:** Vicki Stieha, Brittnee Earl, Harrisen Hagens, Meagan Haynes, Amy Ulappa, Laura Bond, Julia Thom Oxford

**Affiliations:** ^1^Department of Organizational Performance and Workplace Learning, Boise State University, Boise, Idaho, United States; ^2^Center for Teaching and Learning, Boise State University, Boise, Idaho, United States; ^3^Program Evaluation and Research Lab, Boise State University, Boise, Idaho, United States; ^4^Department of Biological Sciences, Boise State University, Boise, Idaho, United States; ^5^Biomolecular Research Center, Boise State University, Boise, Idaho, United States; ^6^Center of Biomedical Research Excellence in Matrix Biology, Boise State University, Boise, Idaho, United States

**Keywords:** active learning, evidence-based instructional practices, STEM education, student motivation

## Abstract

Much of the research on science, technology, engineering, and mathematics (STEM) students’ motivation measures the relationship between student motivation and academic outcomes, focusing on the student’s mindset. Our mixed-methods research takes a different approach and considers the relationship between student motivation and instructional practices. Teaching practices and student motivation were analyzed simultaneously in undergraduate Biology classes using a self-determination theory-based survey to measure students’ motivation during courses that were observed using the Classroom Observation Protocol for Undergraduate STEM (COPUS), and observation notes were collected to document instructor and student behaviors. Quantitative data were used to differentiate students’ motivational levels, and qualitative data were collected to describe how instructors use specific teaching practices. The results provide a lens into how students’ intrinsic motivation varies alongside the instructional practices and interactions in these classes. We found a correlation between higher levels of student motivation in interactive lectures and student-centered teaching profiles. This study highlights how the same practice can be implemented by multiple instructors with varying student motivation scores, pointing out the importance of fidelity to evidence-based instructional practice methods. The results of this study are discussed in the context of published empirical studies examining evidence-based instructional practices that are conceptually supportive of autonomy, competence, and relatedness. Active learning practices observed in this study correlated to positive learning outcomes are discussed and may serve as a guide for instructors interested in implementing specific active learning practices. Recommendations for instructors and departments that are interested in flexible methods to monitor progress toward active learning practices in biology and other STEM disciplines by combining the COPUS and self-determination survey results are presented.

**NEW & NOTEWORTHY** This study uses a novel combination of instruments to describe students’ intrinsic motivation in response to teaching practices. Findings demonstrate that active learning methods may support higher student motivation. Recommendations drawn from the study include using a variety of active learning methods, using evidence-based instructional methods with fidelity, and monitoring the students’ affective response to those methods. Alignment of active learning practices to the components of self-determination may result in higher quality student motivation in science, technology, engineering, and mathematics (STEM) courses.

## INTRODUCTION

Research about science, technology, engineering, and mathematics (STEM) students’ motivation often measures the relationship between student motivation and academic outcomes (1–4), in contrast to the relationship between student motivation and the instructional practices used in courses. We investigated teaching practices and student motivation in undergraduate Biology classes using a self-determination theory (SDT) (5, 6)-based survey to measure students’ motivation during courses that were observed using the Classroom Observation Protocol for Undergraduate STEM (COPUS) (7) and considered the fidelity to evidence-based instructional practice (EBIP) methods among instructors (8).

Nationally, students report a loss of motivation to pursue STEM as a result of their academic experience, which may lead to their departure from STEM degree programs. It is not that they are failing out, but that classroom culture, teaching quality, and the curriculum can be demotivating (9). Yet, these causes of attrition may be ameliorated if instructors implement EBIPs for active learning and other practices that are associated with student’s intrinsic motivation to remain engaged in STEM. Additionally, evidence exists that the core issues in STEM education have an outsized impact on students of color. The percentage of students of color who are negatively affected by their STEM curriculum is 8% higher than for white students (10). Scholarly research demonstrates that strengthening teaching practices and the classroom environment through EBIPs and inclusive practices yields higher rates of student persistence and benefits all students, including underrepresented students in STEM (11–13). These practices also convey a sense of belonging to students (12). The research we present here is empirically aligned with the conditions for effective and academic thriving to cultivate success for all students in STEM classes.

A compelling body of research has demonstrated a correlation between active learning and student persistence (13–18). Research demonstrates that active learning is associated with bolstering students’ psychological processes such as confidence, self-efficacy, and/or sense of belonging (19–23), all of which are associated with motivation in STEM (24–27) and all of which positively impact persistence in STEM. Motivation for learning can be supported by the instructor and the context surrounding learners. Rather than shaping behaviors through rewards and punishment (28), motivational support can create the conditions for students to be self-determined (29).

Incorporation of EBIPs by instructors is key for student persistence in STEM fields (30, 31). Although measuring instructor motivation to change their teaching practices is beyond the scope of this research, providing feedback to instructors regarding their teaching can guide professional development toward EBIP adoption (30, 32). STEM education researchers have demonstrated the efficacy of tools developed to measure and describe EBIPs in STEM. These systematically administered protocols (33–35) provide a picture of active teaching and learning practices that are designed to foster greater adoption of EBIPs. Observation protocols alone, while having strengths, may not capture the differential impact of how particular teaching and learning activities are perceived by students.

The current study emerges from a desire to describe the teaching and learning behaviors in a classroom alongside the students’ affective processes in a systematic manner. To do so, we used two instruments in conjunction: the Classroom Observation Protocol for Undergraduate STEM (COPUS) (35) to measure the level of active teaching and a self-determination theory (SDT) (5, 6)-based instrument to measure students’ motivation in these observed courses. Using both instruments together provides a more complete picture of how the students perceive their experiences of competence, relatedness, and autonomy as well as the instructional practices that support these. COPUS profiles generated through the COPUS Analyzer (36) were augmented with qualitative observation notes to capture the behaviors of the instructor and the students beyond the COPUS code. Results help to answer the research question: to what extent do didactic, interactive, and student-centered classrooms support greater motivation among STEM students?

### Background

This study leverages self-determination research (5, 6), which explains that three psychological phenomena (competence, relatedness, and autonomy) contribute to motivation. Self-determination theory posits that an individual’s quality of motivation is partially dependent on the social and contextual supports for the three motivational components. In the case of STEM teaching, the instructor plays a central role in establishing the conditions for student motivation through instructional practices, curricular design, and classroom culture. This study is framed within the instructional approaches that are widely used in STEM teaching and the research that substantiates their outcomes as they relate to perceived competence, autonomy support, and peer-relatedness. The more a person perceives that these three motivational components are met in pursuit of a goal, the higher their motivational quality will be to pursue that goal (5, 6).

The three motivational components competence, autonomy, and relatedness are described here. Competence refers to the basic need to experience the self as capable of the task at hand (5, 6). When students feel they are capable of succeeding, their perceived competence, or sense that they have what they need to succeed in a course, increases. Competence-supportive teaching strategies are knowledge focused; they include elements of course design, content presentation, and discrete practices during a class. Many EBIPs are conceptually linked to students feeling like they can “operate effectively” and experience “mastery” in the classroom context (6). Autonomy refers to having a choice rather than acting only in response to demands; autonomy results from being empowered (6). Autonomy support is increased when students are provided with the necessary content and structure that will enable them to problem solve and work through challenges (37). It is decreased when student participation and discussion are limited or coerced (38). Importantly, autonomy is not to be confused with independence, but rather it is a relational component of motivation. Relatedness refers to experiencing connection or identification with subject matter as well as feeling recognized and connected to others (6, 39). Students perceive a greater sense of relatedness in a course when they feel like they are cared for, they belong, and they can contribute. While relatedness is measured as a perception by the student, instructors can foster relatedness through practices that help students feel like they belong, that their identities are represented in the course, and that they find content or experiences in the course that are aligned with their values or commitments.

EBIPs have been directly correlated to self-determination in STEM (24, 37, 38, 40–43). Additionally, competence, autonomy, and relatedness outcomes can be correlated to inclusive and active learning practices. Previous studies have focused on outcomes such as academic gains (44–47), belonging (39, 48, 49), self-efficacy (2, 50), and professional identity (49, 51). In this study, we also measured effort, as it is related to motivation. The results of this study include the identification of specific instructional practices that correlate to higher self-determination scores such as questioning during lectures, minilectures, specific discussion approaches, course design, formative/summative assessment, presentation practices, in-class practice, and interactions that foster a positive classroom environment. These are clustered based on their purposes in a STEM course.

## METHODS

The mixed methods research design of this study combined SDT-based survey results with COPUS teaching profiles. Descriptive notes taken during the COPUS observations added detail about the observed teaching and learning activities. Given our research question, these mixed methods are warranted, and we present both quantitative and qualitative data sets concurrently (52). Participants, survey, and COPUS methods are described. This research received institutional review board approval (001-SB19-242). Participation by students was voluntary and responses were anonymous. Written informed consent was received from every participant.

### Participants

The study took place in a medium-sized (∼24,000 students) public, metropolitan research institution. It focused on four core biology courses and all seven faculty members who teach them. The faculty teaching experience ranged from early career to over 25 yr, and all had taught these courses multiple times. Each instructor taught only one course section. Instructors were identified by the notation INST A through G to reduce the risk of identification. Of the four courses, two were lower division and two were upper division. All were undergraduate lecture-based courses, and three had associated laboratories. Two of the courses (one lower and one upper division) were semester-long courses taught in two 8-wk halves by different instructors; the pairs have shared these courses for multiple semesters, and each specializes in the content area for the half they teach. Because the students in split courses were surveyed twice, one for each professor, we treated these data as repeated measures for the survey (discussed below) and for the COPUS we treated them as independently taught (35, 53). Table 1 summarizes course descriptors, enrollment, and instructor experience. The student respondents to the SDT-based survey were enrolled in these courses and are described in results.

**Table 1.** Demographic data for course/instructor combinations

Instructor Code	Course Level	Course	Single/Split Course	Student Enrollment	Faculty Experience Years
A	Upper	1	Split	90	10
B	Lower	2	Split	158	10
C	Lower	3	Single	95	25
D	Lower	3	Single	140	15
E	Lower	2	Split	158	25
F	Upper	4	Single	121	5
G	Upper	1	Split	90	5

Course level indicates upper or lower division. Course indicates which of the 4 courses an instructor taught. Single/split course indicates if the course was taught by a single instructor teaching the full semester or 2 instructors splitting the course, each teaching half a semester. Student enrollment indicates the official registration count for the course. Faculty experience is rounded to the nearest 5-yr increment.

### Self-Determination Theory Survey Data Collection and Instrumentation

To measure motivation, we administered a survey instrument with 15 items. Students were asked to use their own device to complete the instrument via Qualtrics during their laboratory class, except for students in INST F, in which students were surveyed during the lecture section as that course did not have a separate laboratory. If students were absent during survey administration, they did not complete the instrument. There were no incentives offered, and no penalty if students did not complete the survey. The instrument was administered at the conclusion of each instructor’s content area in the split courses (*weeks 7* and *14*) and at the end of the single instructor courses (*week 14*). Students were invited to provide their unique student identifiers, which were used to collect their demographic information.

The instrument was designed based on items included in the Intrinsic Motivation Inventory (IMI) (54) and the Learning Climate Questionnaire (LCQ) (37, 55). The IMI includes seven subscales, although it is rare that all scales are used; the authors recommend that researchers choose the subscales that are relevant to their project (54) and assess them for reliability (e.g., factor analysis). We selected autonomy support questions from the LCQ as they pertain to the social and instructional context established for learning and the competence, effort, and peer-relatedness subscales from the IMI as predictors of greater intrinsic motivation (54). In addition to the three motivational components (competence, relatedness, and autonomy), we included effort, as recommended, from the IMI scale (54) and since it has been positively correlated with student identification as a STEM professional (56) and other relational environments in STEM (40, 56). The original instruments were designed using a seven-point scale ranging from “not at all true” to “very true” (IMI) or “strongly disagree” to “strongly agree” (LCQ). The original IMI uses a midpoint of “somewhat true” whereas the LCQ uses a midpoint of “neutral.”

We developed a four-point scale using “not at all true” (1), “somewhat true” (2), “mainly true” (3), and “very true” (4) to blend the scales. We eliminated a neutral center point as a choice since the scale sought recipients’ opinions about how true the statement was for them, which removed the need for having a midpoint (e.g., “neither true nor untrue”), and three to four categories have proven sufficient to express opinions (57). Further, an ambiguous midpoint has also been identified as a “dumping ground” and satisfies social desirability, particularly among college students (58). As recommended (56), we contextualized the items by using phrases such as “in this course” or “during this course.” For example, a competence-related question reads, “During this course I felt very capable of learning the material” and an autonomy support question reads, “My professor gives me confidence in my ability to succeed in this class” (see Table 2 for all instrument items). Rather than simply assessing each subscale for reliability, it was necessary to validate the instrument as a newly developed measure because two previously validated scales were combined to create the survey instrument and the selected subscales were further altered to fit the context of this research. We verified the four constructs using an exploratory factor analysis (EFA) (59, 60) and calculated an ordinal alpha (61, 62) to provide internal consistency of the identified constructs.

**Table 2.** Exploratory factor analysis with item wording

	Self-Determination Theory-Based Survey Items	Rotated Component Loading			
		1	2	3	4
Component 1: student perceived competence*					
24.	I felt able to meet the challenge of performing well in this course.	*0.86*	−0.03	0.06	0.08
28.	I am able to achieve my goals in this course.	*0.83*	0.07	0.03	0.01
30.	During this course I felt very capable of learning the material.	*0.95*	−0.05	−0.06	0.00
32.	I feel confident in my ability to learn the material in this course.	*0.96*	−0.07	0.00	0.00
*Component 2: autonomy support*†					
40.	My professor encourages me to formulate my own ideas.	−0.05	*0.85*	−0.09	0.06
42.	My professor gives me confidence in my ability to succeed in this class.	0.28	*0.69*	−0.01	−0.06
52.	I feel a lot of trust in my professor.	0.03	*0.81*	0.03	0.01
43.	I feel my professor cares about me as a person.	−0.18	*0.92*	0.08	0.03
Component 3: peer-relatedness*					
46.	I’d like the chance to interact with the people in my course more often.	−0.15	−0.11	*0.68*	0.15
48.	It is likely that a person in my course and I could become friends if we interacted a lot.	0.16	−0.09	*0.80*	−0.06
50.	During this course I felt a connection with other students.	−0.02	0.15	*0.80*	−0.01
54.	During this course I felt I bonded with other students.	0.01	0.06	*0.83*	−0.06
Component 4: effort*					
34.	I put a lot of effort into this course.	−0.03	0.06	0.00	*0.85*
38.	It was important to me to do well at the activities in this course.	0.10	−0.01	0.03	*0.80*

*n* = 658. Rotation method: Promax with Kaiser normalization. Component loading >0.30 is in italics. Items are identified with survey identification numbers. *Survey components adapted from the Intrinsic Motivation Inventory scale (54). †Survey components adapted from the Learning Climate Questionnaire (55).

After removing surveys with incomplete or missing subscale responses, a composite score was generated for each student by calculating the mean score of the items in each subscale. Descriptive statistics were produced based on these composite scores. We used one-way ANOVA to test for differences among instructors on each scale construct. As some students were enrolled in multiple courses included in the study, these students completed the instrument more than once and were therefore subjected to survey response bias. To account for inconsistent repeated measures, only a student’s first survey response was included in the analysis. We used the unique student identifiers to determine who provided repeated responses for this model; it is important to note that the responses of students who did not provide this information (13.7%) were treated as unique students in our statistical model. However, based on identifiable records, less than 2% of the students were dual enrolled in classes that participated in this project. Therefore, while 13.7% of records were not identified, it is not likely that more than 2% of the surveys used for analysis were repeated measures. If a significant difference was identified between instructors for a construct, a post-hoc Tukey’s test was conducted to determine statistically significant differences in construct mean scores between each pairwise instructor combination, with mean score differences considered significant when the adjusted *P* value was less than 0.05.

### COPUS Observation Data Collection

In addition to the survey, an expert observer, administered the COPUS (35) using the generalized observation and reflection platform (GORP) to collect qualitative and quantitative data for these courses. COPUS observations, which involved coding each faculty and student activity per 2-min period (35), were recorded simultaneously with observation notes taken to provide additional details about the classroom activities and teacher/student behaviors observed. The observer comments were recorded with an eye toward the way in which practices were implemented during the observations. Total counts of observations per instructor for Fall 2018 to Fall 2019 are shown in Table 3. The 8-wk split courses were taught by INSTs A/B/E/G and were observed at the middle and end of the 8-wk sections by a single expert observer to balance reliability with available resources while ensuring consistency (63). The single instructor courses (INSTs C/D/F), taught in traditional 16-wk semesters, were observed at the beginning, middle, and end of the semesters. The COPUS protocol generates subject-specific (faculty/student) and categorical (defined COPUS categories) observations that can be rapidly and reliably conducted (64) without requiring recording or videography (1). Reliability was further strengthened as these instructors were observed multiple times over the semesters by the same observer. Each observation was scored independently.

**Table 3.** COPUS Teaching Profiles of Instructors A–G

Cumulative COPUS Profile Results (Fall 2018 to Fall 2019)				
	COPUS Profile			
Instructor	Didactic	Interactive Lecture	Student Centered	Total Observations
A	1	4	1	6
B		4		4
C	2	4		6
D	3	1	2	6
E	4			4
F	3			3
G			2	2
Total	13	13	5	31

Total count of observations in each Classroom Observation Protocol for Undergraduate Classroom Observation Protocol for Undergraduate Science, Technology, Engineering, and Mathematics (COPUS) profile per instructor for Fall 2018 and 2019.

Using the COPUS Analyzer, teaching profiles were generated, categorizing the type of teaching occurring for each observation (36). Three COPUS teaching profiles were generated from the COPUS Analyzer: didactic, interactive lecture, and student centered (36). Didactic classes were characterized by lecturing 80% or more of the time with minimal question and answer. Interactive lecture classes were mainly lecture based with some group activities or clicker questions. Student-centered profiles were characterized by learner-centered strategies being used for most of the instructional time (36). Profiles are presented in results.

### COPUS Descriptive Notes Analysis

The expert observer described practices using a consistent shorthand to summarize behaviors and interactions. The descriptive notes complement the COPUS coding and contextualize faculty and students’ actions and document nuances (e.g., manner in which a teaching practice is conducted, topic, connection to other observed segments, questions that arise, and estimated numbers of people interacting) that are not fully captured by COPUS codes. The supplemental notes allowed for deeper insights into the teaching taking place because COPUS captures many, but not all, aspects of teaching (65). Approximately 33.3% of the 2-min increments included supplemental notes; notes were not added if the interaction was fully explained by the COPUS codes. For example, a segment of strict lecture can be fully captured for both the instructor and the students using the COPUS codes (35). However, the use of instructor talk (e.g., stories, personal examples, or humor) or props is not otherwise captured within the COPUS codes and therefore was described in the notes. Similarly, descriptions of students’ actions (e.g., pairing up after being asked or remaining separated, visible signs of engagement, laughter, nature of questions) were noted as were the nuances pertaining to the EBIP application.

Teaching practices were captured in the descriptive notes. Two researchers applied a priori codes using the SDT constructs (perceived competence, autonomy support, and peer-relatedness) in the following manner. First, the described practices were compared to the scholarly literature in which EBIPs were defined and/or student outcomes were reported. We coded the practices, based on the conceptual agreement between the EBIP (practice description and/or outcome) and the SDT construct definitions. The researchers reached a consensus throughout the coding process. We described an instructor’s personal stories from the biology field as “real-world examples” when they were explaining a concept’s contemporary applicability. The practice was then coded as “competence supportive,” as research demonstrates that new concepts are demystified because real-world examples help learners apply prior knowledge to new challenges (16, 17, 48, 66–68).

Asking questions and answering questions were coded independently in the COPUS protocol for both the instructor and students; the quality or nature of the questioning practice was not captured through COPUS. However, the supplemental notes captured nuances in practice and differentiated three types of questions and answers (Q&A). Asking and answering rapid fire questions (Q&A), which is not an EBIP, is differentiated from Q&A with wait time for student responses and from Q&A with extended discussion, both of which are EBIPs (see Table 6).

We did not code observed practices to effort as this is not one of the basic needs and cannot be objectively observed. We did, however, include effort data in relation to the SDT-based survey data and classroom practice descriptions in the results and discussion.

To support conducting qualitative and quantitative data analysis concurrently, we marked the observed practices bimodally (observed or not) for each INST (52). We have also drawn on the descriptive notes in our findings to illustrate practices that didactic, interactive lecture, and student-centered teaching profiles use to support SDT-based outcomes.

After coding and analysis concluded, we generated a mixed-methods report and shared it with the faculty for participant checking (113). No revisions were made as a result; however, faculty shared their appreciation for the additional detail provided by demonstrating the intersection between SDT variables, teaching practices observed, and COPUS profiles.

## RESULTS

### COPUS Analyzer and Observation Data Results

COPUS observation resulted in teaching profiles for each instructor, categorizing the type of teaching occurring for each observation. Three COPUS teaching profiles were generated from the COPUS Analyzer: didactic, interactive lecture, and student centered (36). Didactic classes were characterized by lecturing 80% or more of the time with minimal question and answer. Interactive lecture classes were mainly lecture based with some group activities or clicker questions. Student-centered profiles were characterized by learner-centered strategies being used for most of the instructional time. The resulting teaching profiles are presented in Table 3. During this study, most instructors’ observations fell into the same COPUS profile across all observations. Teaching observations for INSTs A and D were categorized in all three profiles, while INST C was categorized in two of the three profiles; didactic and interactive lecture. Variation across two teaching profiles is not unusual, but observations across all three profiles is atypical; 9% of COPUS observations vary that widely (36). INSTs E and F were recorded as didactic teaching across all observations, INST B was recorded as interactive lecture, and INST G demonstrated student-centered teaching.

### Self-Determination Theory-Based Survey Results

The total student population for these classes was 852; 669 surveys (78.5%) were returned, of which 491 were used in the analyses (178 were dropped due to one or more missing responses on subscale items, incomplete course information provided in the response, or a student completing the survey more than one time). The demographics of this subset included: 59.8% female, 67.2% STEM majors, and 26.6% underrepresented minorities (114). There were 13.2% freshmen, 37.1% sophomores, 29.4% juniors, 18.7% seniors, and a few (7) postbaccalaureate level students.

A scree plot was generated to identify the most likely number of latent constructs being measured by the modified instrument; the eigenvalues produced in the scree plot indicated that four such constructs existed (λ > 1) (Table 2). Using a Promax rotation and specifying a four-subscale model, an EFA separated all items into their original subscales with loadings higher than 0.50. One item from the autonomy support construct (“My professor makes sure I really understand what I need to do to do well in this class”) cross loaded with the competence construct (0.38) and was removed from the analysis. Using a Promax rotation and specifying a four-subscale model, a second EFA was conducted on the modified data set with the cross-loaded item removed. This modified model did not improve the total variance explained (both the original model and the modified model explained 61% of the total variance) but improved fit indexes and met proposed EFA standards (115), root mean squared error of approximation ≤0.05, and Tucker-Lewis index ≥0.95.

Using ordinal alpha, we found that the constructs for the four competence, four autonomy support, four peer-relatedness, and two effort items had generally high reliability (α = 0.86, 0.89, 0.79, 0.62 respectively). Pearson’s correlation for the survey constructs demonstrated that the highest correlation existed between competence and autonomy support (*r* = 0.64, *P* = 0.001). The other correlations, which are shown in Table 4, were substantially lower.

**Table 4.** Self-determination theory construct mean scores, SD, SE, and Pearson’s correlations for survey constructs

	Pearson’s Correlation						
	Mean	SD	SE	Competence	Autonomy Support	Peer- Relatedness	Effort
Perceived competence	3.03	0.795	0.036				
Autonomy support	2.96	0.782	0.035	0.638			
Peer-relatedness	2.58	0.770	0.035	0.328	0.434		
Effort	3.33	0.623	0.028	0.339	0.344	0.214	

The two-item effort construct demonstrated the lowest reliability. We recognize that a two-item factor is not preferred and that the variance from this subscale is being pushed to the other factors. However, theory and previous scale validation indicate that this two-item factor exists. Removing the two-item factor only marginally improved the overall variance explained (∼1%). Theory suggests that this construct is needed (54); therefore, we retained the two-item factor while acknowledging this as a limitation of our research.

Table 5 provides self-determination theory-based survey results for each instructor. Instructor comparisons identified three of the four constructs that showed significant differences among instructors (peer-relatedness did not). Mean scores for students’ perceived competence were significantly higher for INSTs A/C/D/G compared to INSTs E/F. The mean scores for autonomy support were statistically different between INSTs A/C/D/G and INST E; additionally, INST D was also statistically different from INST F. Comparing the means for the effort scores, nearly all instructors (INSTs A/C/D/E) differed significantly from INST F. The effort scores for INST F, an upper division course, were lowest. Greater agreement was evidenced in the lower division courses for the effort items (“I put a lot of effort in this course” and “It was important to me to do well in the activities in this course”). The model for peer-relatedness did not reveal significant differences between the INSTs. Only instructor comparisons that were statistically significant (*P* < 0.05) are shown in Table 5 Survey results indicated that statistically significant differences existed between composite subscale scores. Given these differences, we looked further into instructional practices. The COPUS Analyzer results and observational data were triangulated with the SDT motivational scale results.

**Table 5.** Self-determination theory-based survey results for each instructor

		Perceived Competence		Autonomy Support		Peer-Relatedness		Effort	
Instructor	*n*	Mean	SD	Mean	SD	Mean	SD	Mean	SD
A	60	3.24	0.768	3.05	0.699	2.61	0.911	3.29	0.588
B	120	2.97	0.854	2.89	0.765	2.60	0.808	3.35	0.633
C	87	3.15	0.791	2.98	0.773	2.62	0.801	3.42	0.599
D	111	3.14	0.739	3.19	0.746	2.61	0.772	3.39	0.574
E	138	2.61	0.747	2.36	0.787	2.50	0.656	3.32	0.615
F	53	2.58	0.751	2.71	0.829	2.35	0.758	3.01	0.671
G	66	3.27	0.631	3.17	0.646	2.65	0.659	3.31	0.649

ANOVA results	*F* Value	*P* Value	*F* Value	*P* Value	*F* Value	*P* Value	*F* Value	*P* Value
	10.013	<0.001	16.344	<0.001	1.769	0.104	2.977	0.007

Post hoc Tukey’s test*	Instructor Comparison	*P* Value	Instructor Comparison	*P* Value	Instructor Comparison	*P* Value	Instructor Comparison	*P* Value
	A, E	<0.001	A, E	<0.001			A, F	0.047
	A, F	<0.001	C, E	<0.001			C, F	0.005
	C, E	<0.001	D, E	<0.001			D, F	0.007
	C, F	0.001	D, F	0.003			E, F	0.023
	D, E	<0.001	E, G	<0.001				
	D, F	<0.001						
	E, G	<0.001						
	F, G	<0.001						

Study variable mean scores, SD, and grouping significances with one-way ANOVA statistics are shown. Only instructor comparisons that were statistically significant (*P* < 0.05) are shown.

### Self-Determination Theory Constructs Layered onto Observed Practices

**Figure 1.**
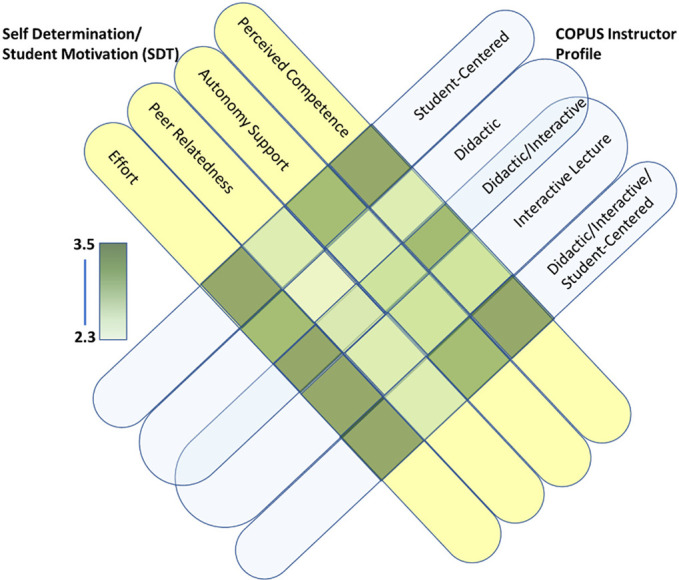
Alignment of the Classroom Observation Protocol for Undergraduate Science, Technology, Engineering, and Mathematics (COPUS) instructor profiles with differences in student motivation data described by 3 self-determination theory (SDT) constructs and effort. Student-centered (INST G), didactic (INSTs E and F), interactive lecture (INST B), didactic/interactive (INST C), and didactic/interactive/student-centered (INSTs A and D) are shown, based on information presented in Table 3. Strong associations between student-centered, didactic/interactive, and didactic/interactive/student-centered COPUS profiles and perceived competence, autonomy support, and effort from student SDT-based survey results (Table 5) are layered onto each profile category as shown. Color intensity scale aligns with mean numerical scores from survey data shown in Table 5.

Many EBIPs can support self-determination and motivational constructs, based on self-determination theory and the references from the literature presented in Table 6. Here, we have observed the teaching practices and measured students’ perceived competence, autonomy, peer-relatedness, and effort. Instructors may approach their courses differently and those differences may contribute to various levels of student motivation. Figure 1 illustrates the alignment of observed COPUS instructional profiles with student motivation outcomes as assessed by our survey.

**Table 6.** Observed instruction practices used by instructors with different COPUS profiles associated with competence, autonomy, and peer-relatedness

Instructional Practice Clusters	E (D)	F (D)	B (I)	C (D/I)	A (D/I/S)	D (D/I/S)	G (S)	SDT Supportive^c^ (References) Competence (C), Autonomy (A), Peer Relatedness (PR)
1. Questioning during lecture/minilecture
Q&A^a,b^	X	X	X	X	X	X		C (66, 69, 70)
Q&A (with wait time)^a,b^	X	X						C (66, 67, 71–74)
Q&A (extended/applied)^a,b^			X		X		X	C, A (51, 75–79)
2. Discussion approaches
Small group and paired discussions			X	X	X	X	X	C, PR (46, 80)
Whole class discussion^b^		X			X	X	X	C, PR (37, 76, 81)
Student report multiple responses^b^			X				X	C, PR (37, 46, 66, 76)
3. Course design
Priming and scaffolding	X	X		X		X	X	C, A (48, 51, 82)
Explicitly stated learning outcomes					X		X	A (48, 51, 67, 68)
Transparency course design (includes setting expectations)					X		X	A (67, 83, 84)
4. Formative/summative assessment					
Polling (raised hand or via clickers^b^)	X		X			X	X	C (85,86)
Quizzes^b^	X		X					C, A, PR (45, 51, 87, 88)
Think-pair-share			X	X			X	C, A, PR (46, 89–91)
Formative assessment (minute paper/muddiest point)				X			X	C, A (47, 90, 92)
Concept mapping						X		C, A (90, 93)
In-class group worksheets^b^			X			X	X	C, PR (45, 49)
5. Presentation-related practices
Real-world examples		X	X			X		C, A (16, 17, 48, 66–68, 71, 94–96)
Multiple representations of content (includes demo video^b^)		X	X	X		X		C, A (66)
Use of humor	X	X			X	X		A (48, 97)
Professor demonstrates humility/vulnerability					X		X	A (98, 99)
Storytelling	X	X	X			X		C, A (48, 66–68)
Peer instruction							X	C, PR (8, 89, 100)
6. Individual, paired, and group practices					
Individual work time^b,d^	X		X			X	X	C (98, 99)
Group work time^b,d^			X	X	X	X	X	C, PR (101, 102)
Moving, guiding/guided instruction^b^			X	X		X	X	C, A (48, 51, 76)
One-on-one interactions^b^			X	X		X	X	A, PR (103–107)
7. General interactions
Professor appears available	X		X	X	X			A (44, 105, 108–111)
Using students’ names							X	A, PR (48, 66, 71, 83)
Connections to student interests; offer research opportunities					X	X		A (42, 48, 66, 103, 105, 109, 112)

Different Classroom Observation Protocol for Undergraduate Classroom Observation Protocol for Undergraduate Science, Technology, Engineering, and Mathematics (COPUS) profiles [didactic (D), interactive lecture (I), student centered (S)] are shown. ^a^Asking questions and answering questions are coded independently in the COPUS protocol for both the instructor and students. We have combined these COPUS codes to reflect the reciprocal nature of questions and answers (Q&A). ^b^Codes that are derived from COPUS. ^c^Self-determination theory (SDT) construct alignment perceived competence (C), autonomy support (A), peer-relatedness (PR). ^d^Individual and group work time are included under “individual, paired, and group practices”; however, individual and group work time are separate codes in the COPUS protocol.

To analyze specific practices, we aligned the individual practices with student motivation and SDT constructs that have been shown to be supported by the individual practices, based on previously published work. We documented whether or not each individual instructor used each teaching practice from the descriptive notes. We clustered the specific practices into categories, based on the descriptive notes and COPUS results into seven groups. These clusters of practices were as follows:

1)  Questioning during lecture or mini lecture (Q&A; Q&A with wait time, Q&A extended/applied);
2)  Discussion approaches (small group and paired discussion, whole class discussion, report out by student groups);
3)  Course content and design elements (priming/scaffolding, explicitly stating learning outcomes, transparency in course design);
4)  In-class formative or summative assessment (polling, quizzes, think-pair-share, minute paper/muddiest point, concept mapping);
5)  Presentation-related practices (real-world examples, multiple representations of content including demonstration videos, professor’s demonstration of humility or vulnerability, use of humor, storytelling, peer instruction);
6)  In-class practice and application (individual and group work time with or without worksheets, professor moving and guiding, and using one-on-one interactions during student work time); and
7)  General interactions (professor appears available, using students’ names, connections to student interests, and sharing research opportunities).

We separated three types of Q&A occurrences in light of the previously published scholarly literature and coded the SDT motivational scale alignment based on the practice descriptions and outcomes cited in the literature for each (see Supplemental Table S1 for further illustration of the qualitative coding process). We did not code observed practices to effort, as this is not one of the self-determination motivational constructs and cannot be objectively observed. We did, however, include effort data in relation to the SDT-based survey data and classroom practices descriptions in the findings and discussion.

### Questioning During Lectures or Mini-Lectures

We observed that all but INST G used a rapid-fire questioning approach. The didactic instructors (INST E/F) also added wait time for reflection before a shout-out response. By contrast, interactive lecture and student-centered instructors used Q&A practices that incorporate time for reflection and those that open up extended discussion either as a whole class or in small group activities, which have been shown to be supportive of competence and autonomy by previously published studies, whereas didactic instructors relied heavily upon rapid-fire questioning. Only INST B/A/G use the critical thinking intensive strategies for questioning.

### Discussion Approaches

The interactive lecture and student-centered instructors INSTs A/B/C/D/G all used small group and paired discussion in conjunction with lecture or minilecture and Q&A approaches; the didactic INSTs E/F did not. Whole class discussion was also used by INSTs A/D/F/G across the spectrum of profiles in conjunction with lecture or minilecture; however, INST F used 10 two-minute segments, whereas INST D/G engaged in whole class discussion 16 times. Additional formative assessment practices were used by INST B/C/D/G. The only use of concept mapping was observed in INST D as a readiness activity. INST C/G used minute papers. INST C used them to start a class, connecting content to a prior session, and INST G asked students to work through handout questions during class, during which there were pauses to clarify and interpret questions. INST G also asked students to write reflectively about course content using a minute paper in this study.

### Presentation-Related Practices

The presentation-related practices observed in this study were clustered into five categories, three of which have been shown to be supportive of both competence and autonomy (real-world examples, multiple representations of content, and storytelling) from previous studies, and two of which have been shown to support autonomy (use of humor and professor’s demonstrating humility or vulnerability). INST F/B/D integrated real-world examples into their lectures. INST F/B/C/D also used multiple representations of content in their lectures. These strategies have been shown to foster greater mental engagement in lectures and can help contextualize abstract content for students who may then be able to connect their own lived experience to the phenomenon being discussed as shown by published studies (16, 17, 48, 66–68, 71, 94–96).

The use of humor and storytelling has been shown to enhance competence and increase course engagement by making the content relatable to students. Humor was used by INST E/F/D/A, all of whom relied heavily on lecture in their classes. Joking or humor was used throughout INST D’s classes and occurred more than in the other classes, although not all the jokes were content related. INST E/F/B/D enhanced the material presented in class such as sharing stories from the field or their experience pipetting DNA during graduate school.

In our study, peer instruction, when students address the full class regarding course content, was only used by INST G during these observations. The single instance followed questions posed by the instructor and subsequent small group discussions with the expectation that individuals would explain responses to various questions on a worksheet. The peer instruction was followed by a mini-lecture with additional clarification.

### Individual, Paired, and Group Practices

Individual and group work time for students to work on problems or course-related content during the class was captured in the COPUS codes. In this study, group work was observed for INST A/B/C/D/G, all of whom demonstrated aspects of interactive lecture or student-centered instruction profiles (Table 3). INST B/D/E/G incorporated individual work time into their courses; this code was only used when an instructor specifically asked students to work individually on a problem in class. INST A used organized groups and asked students to work together to discuss a problem in conjunction with lecture and whole class discussion. While INST G required students to work on handouts or problems in groups, INST B permitted students to decide whether to work individually or in small groups on guided worksheets, an example of process-oriented guided-inquiry learning that has been shown to support competence and autonomy (116). As the students worked, INST B moved throughout the room and guided the individuals or pairs in their work. Of the instructors who used individual and group work time, only INST A did not also use moving and guiding or one-on-one interaction during those activities.

One-on-one interaction is a COPUS code for the instructor’s activity during which they engage in a discussion with one or more students in close proximity to them rather than doing so in front of the whole class. While INSTs B/D/G used one-on-one in conjunction with moving and guiding, INSTs C/D also used it separately. INST C engaged in a brief discussion with one student when reviewing the muddiest point paper. INST D had a conversation with a student about softball that was overheard by the observer during an activity that also included moving and guiding. These observations point to the way that faculty can connect on an individual level with their students during these activities, and previously published works indicate that this type of activity is supportive of building rapport (103, 108) and supporting autonomy.

### General Interactions

Self-determination theory literature has shown that general interactions such as instructors appearing available at the beginning of class while doing administrative tasks and using students’ names, particularly in a large class, help foster a positive classroom culture and may send a message of caring from the professor to the students (66, 109). In our study, INSTs A/B/C/E were observed to carry out these practices. The observation notes for INST G documented name use (“instructor is using students’ names [while calling] on them”), which has been shown to contribute to a positive classroom culture (66, 109). Finally, INSTs A/D were observed extending opportunities for additional support. INST A, for example, invited students to make an appointment, specifically mentioning the desire to “help them be successful in the course.” All of these small moves have been shown by others to support an inclusive classroom (71) and be autonomy supportive. INSTs C/A/D/G were among the higher mean scores for autonomy support in this study (Table 5).

### Effort and Observed Practices

High effort scores suggest stronger student agreement with statements, such as, it is important to do well in the class, and that the respondent put a lot of effort into the class. INSTs A/C/D/E demonstrated statistically significantly different mean scores than INST F; however, no other differences reached significance. The differences between the effort scores for INSTs F (didactic profile), B (interactive lecture profile), and G (student-centered profile) were not statistically significant (Table 5), although the behaviors and activities in their classes clearly differed. INST E had a higher mean score for perceived effort (*P* < 0.023) than INST F, yet they both fell into the didactic profile. INST E used two active learning practices (polling and quizzing) that were not used by INST F, while INST F used multiple representations of content, real-world examples, and whole class discussion during lectures, and INST E’s lectures were only interspersed with Q&A.

**Figure 2.**
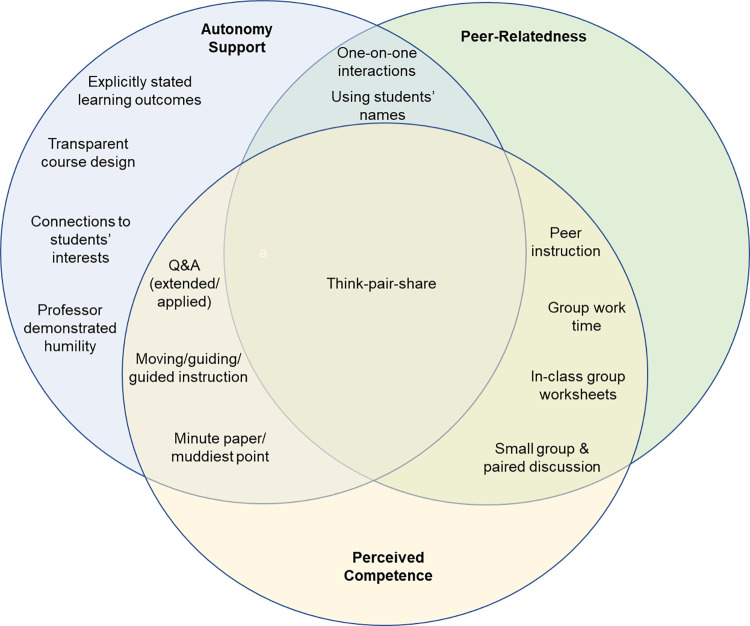
Practices for which theoretical self-determination theory (SDT) support for competence, autonomy, and peer-relatedness align with SDT-based survey measures from this study. Observed instructional practices that correlated with higher student survey scores (Table 5) and align with the previously published results from the literature (Table 6) are grouped with respect to competence, autonomy, and peer-relatedness. In many cases, an individual instructional practice will have been shown to be supportive of more than one SDT component, as illustrated by the Venn diagram. Observed instructional practices from this study that align with theory from previously published works include questions and answers (Q&A; extended/applied), small group and paired discussions, explicitly stated learning outcomes, transparent course design (including setting expectations), think-pair-share, minute paper/muddiest point, in-class group worksheets, professor demonstrating humility, peer instruction, group work time, moving, guiding/guided instruction, one-on-one interactions, using students’ names, and connections to student interests.

Table 6 combines observed classroom practices coded for each instructor aligned with self-determination theory and outcomes from the literature regarding practices that have been shown to be supportive of SDT constructs. We coded teaching practice from the descriptive notes as perceived competence, autonomy, and peer-relatedness supportive based on the scholarly literature. We have included references in the table that provide empirical evidence of the previous outcomes of the specific practices. Table 6, *column 1*, clusters the described practices into categories. COPUS profiles are identified for each instructor based on information presented in Table 3. INSTs A and D were categorized in all three profiles, while INST C was categorized in two of the three profiles; didactic and interactive lecture. INSTs E and F were recorded as didactic teaching across all observations, INST B was recorded as an interactive lecture, and INST G demonstrated student-centered teaching. Figure 2 highlights observed instructional practices that correlated with higher student survey scores from this study (Table 5) and align with the previously published results from the literature. Observed instructional practices that align with theory include Q&A (extended/applied), small group and paired discussions, explicitly stated learning outcomes, transparent course design (including setting expectations), think-pair-share, minute paper/muddiest point, in-class group worksheets, professor demonstrating humility, peer instruction, group work time, moving, guiding/guided instruction, one-on-one interactions, using students’ names, and connections to student interests.

## DISCUSSION

We have reviewed the practices that have been associated with outcomes including cognitive and affective gains and explained how these practices theoretically align with greater self-determination or the components of intrinsic motivation. We note that many EBIPs can be used to support these SDT constructs and that we can both observe the practices and measure students’ perceived competence, autonomy, and peer-relatedness. As we consider the results of this study, we can see that instructors approach their courses differently and that those differences may contribute to various levels of student motivation.

### Self-Determination Theory Supportive Practices

There is a growing body of literature that directly correlates EBIPs to SDT in STEM (24, 37, 38, 40, 43). There is also a sizeable body of research demonstrating competence, autonomy, and/or relatedness outcomes correlated to inclusive and active learning practices even though they do not specifically measure student’s self-determination. Rather, the literature tends to focus on outcomes such as academic gains (44–47), belonging (39, 48, 49), self-efficacy (2, 50), and professional identity (49, 51). Outcomes of practices that are commonly observed in North American universities’ STEM classes (36) that pertain to self-determination components are listed below. We also discuss effort, as it is related to motivation and measured in this study. In doing so, we have clustered practices based on their purposes in a STEM course.

### Questioning During Lecture or Minilecture

Some of the questioning techniques that were observed in this study and the resulting SDT-based survey scores from this study did not align well with our expectations from the previously published literature. Questioning techniques targeting higher order critical thinking (69, 75, 89, 117) have been correlated with better academic outcomes and have been shown to foster the type of challenges inherent in building competence (5, 6). While identification-type questions may support competence, open-ended and problem-solving and higher order questions requiring critical thinking have been shown to bolster autonomy (37, 46, 66, 76). Adding peer interaction fosters stronger academic gains (46, 80, 118), metacognitive growth (86), and relatedness outcomes as well (46, 80, 118), as documented in previously published studies. Reducing volunteer-only responses by randomly calling on students or cold calling may reduce volunteer bias when integrating questions in lectures. Reducing volunteer bias has been shown to increase belonging, thus relatedness, as a greater diversity of voices are heard in the class (70). Further, substantial research has identified the need for listeners to have ample wait time (greater than 3–5 s) to cognitively process and formulate an answer (66, 67, 71–74). In this study, we found that all instructors used Q&A practices that have been shown to support competence and autonomy (Table 6); however, A/B/E/F used more than one practice, and INSTs A/B/G utilized Q&A with extended times for response and with application. Survey results from this study identified INSTs A/C/D/G with statistically higher scores for competence and autonomy (Table 5). Our results support the conclusion that similar practices implemented by different instructors can have different outcomes.

### Discussion Approaches

Active learning practices that enable students to practice ideas in a safe space (e.g., small group or paired discussion) may contribute to lowering student participation apprehension and lead to greater classroom engagement (104) increasing autonomy support. While such engagement has been correlated with positive academic gains (39, 48, 97), it has also been shown to support positive peer connections when conducted in an inclusive classroom (39, 71). Students can build a stronger STEM professional identity through professor interactions (103, 105, 106) and exposure to diverse representation showcasing individuals and ideas in the class and content (19, 119), which suggests that the practice of including many student voices in whole class discussion is aligned to peer-relatedness (37, 76) even as it also supports students’ conceptual understandings (46). A caveat, however, is that free-flowing social interactions have been shown to be far less likely to generate strong relatedness outcomes compared to structured small-group interactions (81). In this study, all instructors with the exception of INST E were observed to practice specific discussion approaches that have been shown to be effective supports of competence and peer-relatedness. INSTs A/C/D/G had higher scores for competence from our SDT-based survey; however, there were no differences between instructors that reached significance for peer-relatedness. We found that the practice of small group and paired discussions and competence scores from our survey aligned with our expectations from the previously published literature.

### Course Design

All instructors with the exception of INST B were observed to include course design practices that have been shown to support competence and autonomy. Survey data from our study indicated that INSTs A/C/D/G had higher scores for competence and autonomy. Effective course design has been shown to increase student engagement and learning (48, 108, 120, 121). Priming, scaffolding (48, 51, 82), communication regarding learning outcomes (48, 51, 67, 68), and explanations of the course structure (67, 84, 122) have been shown to support cognition as students connect different concepts to one another. Students perceive these explanations and structural signals as helpful (48, 51, 82) and as contributing to their ability to understand STEM concepts in their courses (48); thus they have been shown to be both competence and autonomy supportive. Differences between those instructors with higher scores and those with lower scores from our survey data may be explained by the utilization of the practices of explicitly stating learning outcomes and transparency in course design, including setting expectations. These two practices and scores from our survey data align with previously published studies. In contrast, the differences between observed practices and mean scores from our survey may indicate that priming/scaffolding were not as effective as the other practices observed in our study.

### Formative/Summative Assessment

Previous studies have shown that instructors can integrate formative assessments such as muddiest point, minute papers, and concept mapping into classes to positively impact students’ perceived competence and autonomy (47, 90, 92, 93). These practices have been shown to provide timely feedback through which students can gauge their understanding. Recent research comparing the muddiest point to other formative response methods such as oral response and concept mapping indicates stronger academic gains in the muddiest point group (47), while minute papers strengthened students’ preparation for exams (92). Preparation and accountability are also strengthened through regular in-class quizzes, which can be used formatively or summatively to support perceived competence (45). Autonomy is also supported when quizzing is formative and facilitated through electronic response systems or clickers during instruction and the class’ collective responses are visible (45, 51, 87, 88). One particular approach to quizzing, Mazur’s peer instruction or ConcepTest strategy (89, 100), provides strong evidence linking it to competence, autonomy, and peer-relatedness support. Similar to peer instruction, think-pair-share has robust empirical support for multiple SDT constructs and has been correlated with academic gains (46, 80, 118), metacognitive growth (86), and favorable STEM attitudes (15, 43, 107). In this study, INSTs B/C/D/E/G were observed to incorporate formative/summative assessment practices that have been shown to be supportive of the SDT components competence, autonomy, and peer-relatedness. Polling and quizzes were not aligned well with our survey results; however, think-pair-share, minute paper, muddiest point, and in-class group worksheets did show good alignment between the expected outcomes from previously published work and our survey results.

### Presentation-Related Practices

Teaching strategies that help students identify with the subject matter and feel they belong in STEM have been shown to contribute to success and are correlated with academic gains (11, 123) and persistence (25, 43, 56). When presenting content, professors can help demystify new concepts through real-world examples, thereby supporting autonomy as learners conceptualize the concept in light of prior knowledge via the real-world example (16, 17, 48, 66–68). Similarly, offering multiple representations of concepts (e.g., visual, verbal, manipulative) has been shown to support greater comprehension of abstractions (66, 94, 95). Another example of simultaneously supporting autonomy and competence is “instructor talk” (67, 68), during which teachers explain pedagogical choices or demystify concepts by relating them to lived experiences.

Student-centered teaching including faculty approachability (44, 105, 108–111), storytelling (48, 66–68), and higher energy level (48) has been shown to be inherently relational and to foster an empowering class climate (110). A spirit of fun, excitement, and energy are all correlated with student persistence in STEM (48). Humor, when related to the content, has been shown to increase students’ information processing ability as well as persistence (48, 97). Finally, showing vulnerability (e.g., humility or showing that instructors make errors, are not perfect, and do not know everything) (98, 99) has been shown to support a positive culture for discussion; hence, it is relatedness supportive. In this study, we found that all instructors implemented presentation and related practices that have been shown to support competence, autonomy, and peer-relatedness. Alignment between our survey data and specific practices was seen for the practices of the professor demonstrating humility/vulnerability and peer instruction. For other practices, alignment was less clear, as results were mixed and may be explained by different instructors applying similar practices differently with different outcomes.

### In-Class Practice and Application

In this study, we observed that all instructors with the exception of INST F implemented individual, paired, and group practices that have been shown to support competence, autonomy, and peer-relatedness. Providing students with in-class practice time to apply course content helps them develop competence with course concepts and engage with the course materials and their peers increasing perceived autonomy and peer-relatedness. Active lectures can integrate these application experiences, which contribute to intrinsic motivation. Using in-class worksheets, particularly in small groups, has been shown to increase science self-efficacy gains and a sense of social belonging (45, 49). In addition, discourse and inquiry among students can provide opportunities to build student’s perceived competence, autonomy, and relatedness. Even relatively brief peer exchanges have been correlated to stronger problem-solving abilities and positive student attitudes (51, 76). These can be distinct from questioning from teachers and more extended guided discussion. The results of our survey data indicate better alignment with the existing literature for group work time, moving, guiding/guided instruction, and one-on-one interactions.

### General Interactions

Previous studies have shown that interactions that help foster a positive classroom environment and trust are critical for robust learning (99, 109, 124). In addition to building rapport between the instructor and students (99), a positive class culture includes using students’ names (48, 66, 103, 109), as well as learning students’ academic, personal, and professional interests (42, 48, 66, 103, 105, 109). Previous research points to the ways that these practices foster psychological safety and authentic caring, which encourages students to take intellectual risks (99, 125, 126). Thus these practices conceptually align primarily with autonomy support as described above. In this study, all instructors with the exception of INST F were observed to practice general interactions that have been shown to support autonomy and peer-relatedness. The survey results from this study indicated that INST A/C/D/G had higher mean scores for autonomy, indicating better alignment between this study and the existing literature for the practices of using students’ names and connections to students’ interests.

### Perceived Effort

While it is not one of the basic psychological needs, effort is related to the other SDT components (54) and integral to motivational processes (127). Effort has been shown to be dependent on task value and is associated with student engagement (128). That is, if a student perceives the task as something they can do and something that they find worthwhile doing, they will be more likely to exert greater effort (39). Decontextualized learning can be perceived as lacking task value and is associated with lower student engagement (129) and motivation (6). A relationally supportive environment has been shown to be empirically tied to increased student effort (40, 56).

Additionally, there are individual psychological processes that play into effort, which may include distractions beyond the course (127), but these processes can be positively impacted by the course design and the instructors’ actions to help sustain student effort (130, 131). In this study, survey results indicated that INSTs A/C/D/E had higher mean scores than INST F for effort.

### Similar Practices with Different Outcomes

When generally implemented, the COPUS identifies strategies used; it does not detail the manner in which they are used (132). A trained observer can see differences in the look and feel of the classes. As described above, the observed classes applied the same practices quite differently and the mean scores for perceived competence, autonomy, and effort differed. These findings suggest there could be a range of student learning gains, which reflects prior research (120, 133).

For example, all of the INSTs in our study used “asking questions” (COPUS code PQ) and “answered questions” (COPUS code AnQ), and a COPUS analysis would look similar for them in these categories. Yet, the qualitative data highlight important differences demonstrated in the literature to impact motivation. The rapid-fire Q&A, commonly observed in the didactic profiles, is often limited to lower recall or identification questions (134) and maps to competence only. That style contrasts starkly with extended Q&A, which often requires a greater level of critical thinking (69, 79) supporting competence and autonomy. Extended Q&A was also used in conjunction with other active learning practices in INSTs B/C/A/D. Further, while instructors who used active learning practices sometimes used the rapid response Q&A pattern, those who dominantly use it are associated with didactic teaching styles (76). The difference between higher order thinking and lower-order questions may be reflected in the lower perceived competence scores in the didactic-profile classes as an appropriate level of challenge is important for perceived competence (5, 6).

Unlike prior research in which students self-report perceived competence in active learning classes as lower than in didactic classes (133), here the students in the active learning settings had higher competence and autonomy support scores (INSTs A/C/D/G). It is possible that the differential findings can be attributed to studies using student preference survey items (133) versus employing theory-based items. The two highest scores (INST A/G) were also in an upper division course. Prior experience in lower division core courses that use interactive lectures might have acclimated students to active learning in this case, as has been noted elsewhere (133). We see this finding as consistent with findings suggesting that efforts to increase active learning across the curriculum are more effective than solitary faculty efforts (32, 135).

The interactive lecture and student-centered instruction INSTs SDT-based survey mean scores were higher than the didactic INSTs, and they used more EBIPs in conjunction with one another. For example, INSTs A/C/D/G draw uon practices such as using student names and reducing the professor-student divide through moving and guiding or one-on-one interactions, practices that are positively correlated with a more inclusive classroom culture, which supports belonging (71, 109) as well as sending strong autonomy support messages to them. These instructors have the highest autonomy support scores as well. We contrast these activities by only using the appearance of availability as a rapport-building practice (INST E), where the autonomy support score is statistically lower. While the observation notes point to a posture of availability that could support belonging (44, 105, 108–111), it is more passive than other rapport supports and relies on the students to take the first step.

Our findings are consistent with the literature asserting that managing classroom interactions and using EBIPs for collaborative groups fosters greater engagement outcomes for students (136), but lack of fidelity in implementation reduces positive outcomes (8, 67). In our data, the percentage of students working in groups was higher in INST G than for INST C. Whereas INST G intentionally placed students in clusters to support grouping, INST C asked students to work in groups but did not structure the class seating pattern to ensure that students were near peers without moving, as a result, many students worked individually instead. INST B provided the option to choose to work in groups or individually. When directing students into groups, INST G achieved greater engagement in terms of the observable activities in the class.

### Effort and Active Learning

As others have noted, effort questions have a relatively low correlation with behavioral measures, suggesting the need for student input to better interpret the responses (2, 54). Still, some discussion of these findings relative to effort is warranted.

Examining the effort items (“I put a lot of effort in this course” and “it was important to me to do well in the activities in this course”), it is possible that the instructional practices used by the didactic professors did not engage respondents to the degree that students felt a high task value (128). Since we understand that a relationally supportive environment increases the desire to exert greater effort (56), we would have expected to see a larger difference between the effort mean scores when comparing the courses with greater autonomy practices to those with fewer autonomy practices. The fact that INST E teaches an upper division course for a full semester and INST F teaches a lower division split course may also add the complexity of comparing effort scores.

In light of our insights and the literature highlighting the many factors impacting student effort, it is clear that a better understanding of how perceived student effort impacts the outcomes of active learning practices is needed. It is also worth considering how perceived effort and autonomy interact when students are required to complete core courses for their major versus as an elective, as suggested by others (41, 105); however, our data cannot answer that question. Students’ perceptions of effort (high or low) can be experienced differentially (137), and faculty who use active learning strategies can reduce students’ negative perceptions of effort by addressing it directly (133). In short, further interpreting our effort findings without explanation from the student respondents is not possible.

### Conflicting Data Relative to Peer-Relatedness

Our qualitative analysis paints a different picture of peer-relatedness than is evidenced in the SDT-based survey data. Collaborative practices, such as small group and paired discussions, group work time, think-pair-share, and one-on-one, are widely used in the interactive lecture, and in addition, using students’ names and peer instruction was unique to the student-centered profile classes; however, practices that support peer-relatedness were seldom used by the didactic profile INSTs (limited to only whole class discussions and quizzes). Given the qualitative differences described above, we were surprised that there were not significant differences in the peer-relatedness mean scores. It is possible that the question wording may partially explain these confounding data. For example, “I’d like the chance to interact with the people in my course more often” could be answered differently depending on their classroom experiences. If a student did not have the chance to get to know their peers, they might disagree because they have no information on which to base their desire to get to know them better. Whereas if a student experienced a high level of interaction during the course, they could also answer “disagree” because they were content with the level of interaction, having gotten to know their peers. Other questions such as “During this course I felt a connection with other students” and “During this course I felt I bonded with other students” may have been answered differently depending on the classroom experiences and interpretation of the question. Finally, the notion that students’ desire to build friendships beyond the class experience may be attributed to an unconscious bias privileging mainstream middle- and upper-class values, which might not align with those held by nontraditional and underrepresented students currently matriculating (138). Certainly, the EBIP outcomes regarding peer learning are not predicated on becoming friends (“It is likely that a person in my course and I could become friends if we interacted a lot”), but rather working collegially, helping one another to learn the course content.

### Conclusions

The findings of this study add to the body of literature arguing that faculty can leverage EBIPs to improve students’ motivation to persist in STEM disciplines. While encouraging faculty to use practices other than lecture is important, understanding that the way EBIPs are implemented matters when it comes to achieving positive academic and affective outcomes. In this study, the active learning and interactive instructional profiles correlated with higher mean scores on the motivation scale based on our survey results.

A large body of literature points to grade performance associations with active learning (13, 15, 51, 105, 133), but there is far less that helps faculty see the connection between interactive lecture or student-centered teaching and perceived competence, relatedness, and autonomy associated with higher motivation. In particular, our study adds to the work of Vanasupa et al. (43) pointing to evidence that higher quality motivation is associated with active learning practices. The combination of two highly researched tools (COPUS and SDT) effectively demonstrates the range of strategies that instructors in this study are using, which correlate with higher student motivation in their courses.

### Recommendations

Our mixed methods study contributes to filling the gap identified by Stains and Vickrey (8) who comment on the need for more data surrounding the fidelity of implementation for EBIPs. It is even possible to negatively impact students if active learning practices are not used inclusively (139). Future mixed-methods research that integrates qualitative data regarding instructor and student perceptions of EBIPs into combined COPUS and SDT-based survey measures would strengthen our understanding of the critical components of these EBIPs that may impact the environment and experience of students to help them learn, persist, and graduate in STEM fields. These findings may help us develop a fidelity implementation framework (61).

A related research recommendation is to develop SDT-based survey items that more accurately reflect the degree to which students are interacting in ways that help them build a STEM identity and meaningfully connect with peers in their STEM courses. Further, as relatedness also pertains to disciplinary connections, integrating questions that focus on relatedness support via the curriculum might better inform that SDT component. The two items of the effort scale also had a lower reliability than the other constructs and might be confusing to interpret for students. Future research is needed to refine effort-related questions that are more consistent with SDT theory and applicable to active learning in STEM.

Despite the evidence that EBIPs increase student success and persistence in STEM, widespread adoption of these effective practices has been slow. Recent research examining barriers and drivers for EBIP adoption highlights the opportunity to help instructors build on the active learning EBIPs that they are currently using to propel the use by instructors further (30, 31). This would work particularly well for faculty who are inclined to adopt EBIPs and who are in a departmental climate that is perceived as supportive of such changes (140,141), providing information that affords faculty autonomy, competence, and relatedness through the social context for teaching and learning could help faculty adopt and continue to refine their use of EBIPs. Anecdotal comments from the faculty involved in this study noted the helpfulness of seeing the SDT-based survey results in conjunction with their COPUS data and supported their continuing efforts to reform their teaching practices. We recommend using the SDT-based survey and COPUS conducted in concert to paint a more complete picture of faculty practices and student affective responses. Adding notes systematically during the COPUS observations as recommended (7) was highly informative in this study as doing so affords far more detail about the way a practice is administered in various classes.

Finally, faculty developers and departments can use Table 6 and Fig. 2 to identify teaching practices associated with the instructors whose teaching profiles are identified as interactive lecture and active learning profiles and Table 5 to identify instructors with higher SDT-based survey scores. Note that we have included references in Table 6 to support faculty who are seeking empirical evidence of the outcomes as well as selected articles describing the practices as resources toward that endeavor.

### Limitations

Our application of the COPUS included one highly experienced observer; it is possible that two observers with high interrater reliability would generate additional data. The split course structure with each instructor teaching only 8 wk limited COPUS observations to two per INST while 16-wk courses were observed three times each. Finally, the reliability of the effort construct in these data is a limitation as some of the variance from the subscale was pushed to other subscales.

## DATA AVAILABILITY

Data will be made available upon reasonable request.

## SUPPLEMENTAL MATERIAL

10.6084/m9.figshare.26069476.v2 Supplemental Table S1: https://doi.org/10.6084/m9.figshare.26069476.v2.

## GRANTS

This work was supported through National Science Foundation Scholarships in Science, Technology, Engineering, and Mathematics (S-STEM) Grant 1644233. We thank our grant collaborators including Jennifer Forbey and Kevin Feris. Statistical analysis for this research project was supported by the Biomolecular Research Center under National Institutes of Health, Center of Biomedical Research Excellence (COBRE) Grant P20GM109095 and Idaho IDeA Network for Biomedical Research Excellence (INBRE) Grant P20GM103408.

## DISCLOSURES

No conflicts of interest, financial or otherwise, are declared by the authors.

## AUTHOR CONTRIBUTIONS

V.S., B.E., A.U., and J.T.O. conceived and designed research; V.S., B.E., H.H., M.H., and L.B. analyzed data; V.S. and B.E. interpreted results of experiments; V.S., H.H., M.H., and J.T.O. prepared figures; V.S., B.E., H.H., M.H., and L.B. drafted manuscript; V.S., B.E., A.U., and J.T.O. edited and revised manuscript; V.S., B.E., A.U., L.B., and J.T.O. approved final version of manuscript.
